# Reproducibility and relevance in insect-arbovirus infection studies

**DOI:** 10.1016/j.cois.2018.05.007

**Published:** 2018-08

**Authors:** Anthony James Wilson, Lara Ellen Harrup

**Affiliations:** The Pirbright Institute, Ash Road, Pirbright, Woking, Surrey GU24 0NF, United Kingdom

## Abstract

Experimental infections of insects with arboviruses are performed to achieve a variety of objectives but principally to draw inferences about the potential role of field populations in transmission or to explore the molecular basis of vector–pathogen interactions. The design of such studies determines both their reproducibility and the extent to which their results can be extrapolated to natural environments, and is constrained by the resources available. We discuss recent findings regarding the effects of nutrition, the microbiome, co-infecting agents and feeding methods on the outcome of such experiments, and identify resource-efficient ways to increase their relevance and reproducibility, including the development of community standards for reporting such studies and better standards for cell line and colony authentication.

## Highlights

• The design of infection studies reflects relevance, reproducibility and resources.
• We review recent studies of factors affecting the outcome of such experiments.
• Such factors include vector origin, maintenance, infection and detection methods.
• We identify resource-effective ways to increase relevance and reproducibility.

## Graphical abstract

**Figure:**
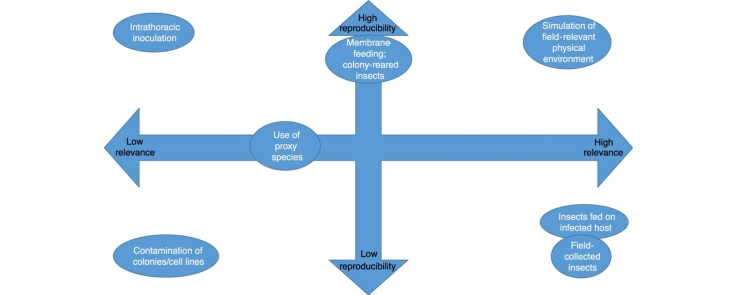

**Current Opinion in Insect Science** 2018, **28**:105–112

This review comes from a themed issue on **Vectors and medical and veterinary entomology**

Edited by **Jason L Rasgon**

For a complete overview see the Issue and the Editorial

Available online 23rd May 2018

**https://doi.org/10.1016/j.cois.2018.05.007**

1877-3435/© 2018 The Authors. Published by Elsevier Inc. This is an open access article under the CC BY license (http://creativecommons.org/licenses/by/4.0/).

## Introduction

Blood-feeding insects such as mosquitoes, *Culicoides* biting midges and sandflies are capable of transmitting a range of important human, zoonotic and veterinary viruses such as Zika virus (ZIKV), Rift Valley fever virus (RVFV) and bluetongue virus (BTV). The proportion of individuals in a population that develop a transmissible infection in response to exposure to an arbovirus is defined as its vector competence. Experimental infections of insects with arboviruses are mostly performed either to draw inferences about the potential role of field populations in transmission, which include but are not limited to their vector competence (either of populations within the current range of an outbreak to inform control strategies [1,2], or of populations beyond the current range of an outbreak as a way to explore the outbreak’s potential for further expansion [3]), or to explore the molecular basis of vector–pathogen interactions [4]. Less commonly, such experiments are also used to study the effects of infection on behaviour [5] and, increasingly, to evaluate modifications intended to render vectors refractory to a pathogen, either via genetic editing [6] or via co-infection with other microorganisms such as *Wolbachia* bacteria [7].

Reproducibility is the basis of the scientific method [8]. Despite this, it is increasingly recognised that a high proportion of experiments published in the peer-reviewed literature are not reproducible [9^•^,^10^]. Partly, this is down to the use of inappropriate statistical analyses to infer significance [11], but it is also attributable to incomplete reporting of methods. There is increasing awareness that factors influencing the components of vectorial capacity are inadequately measured or controlled in the laboratory or reported in the literature, and may differ substantially from factors to which natural populations are subjected. Furthermore, natural populations vary in their susceptibility [12] and colonies established from small populations are not likely to be representative of the global population of the species. This may cause laboratory results, to differ markedly from both those observed in the field and those obtained in laboratories attempting to replicate findings. A related issue is that of relevance: if key aspects of the environment to which natural populations are subjected before, during, and after infection differ from those to which a study population is subjected in the laboratory, then while the resulting study may be highly reproducible, the result may not be relevant to transmission under natural conditions. Whether noted by the original researchers or not, this may not be recognised by later users of the data, for example when used to parameterise transmission models or conduct meta-analyses.

**Figure 1:**
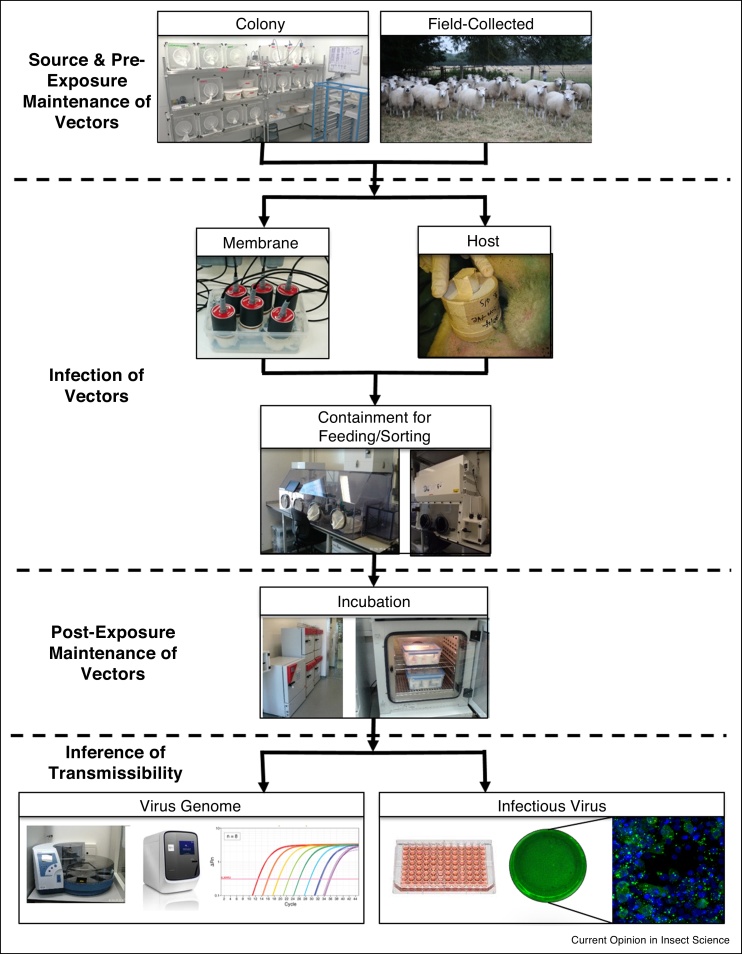
A typical work-flow for an insect-arbovirus infection study and with key decision points illustrated.

The critical factors determining the outcomes of experimental arbovirus-vector infection studies fall into four broad categories: first, the source of the vector population studied, second, how the vectors are maintained before and after infection, third, the method used to infect the vector, and fourth, how infection in the vector is characterised. These four stages are illustrated in Figure 1. In this review, we discuss and evaluate examples of studies that identify factors falling into in each of these categories and their implications for reproducibility and relevance.

## Source of vectors

Laboratory insect–arbovirus interaction studies generally use either colony-reared individuals [1,13] or field-collected specimens [14,15], or more rarely a combination of the two within a single study [16]. The use of colony-reared insects presents several advantages. The first is that large numbers of specimens can be reliably obtained at any time of year, which is a particular advantage for researchers working in areas where the vectors of interest are absent, seasonally absent or occur at low abundance. The second is that the historical environment experienced by the insects is known and controllable, allowing the use of insects of known age, maintenance history, prior pathogen exposure and even genetic diversity, potentially increasing the reproducibility of the experiment. The low levels of heterozygosity found in some colonised populations [17, 18, 19] may also provide increased power to detect trait differences. However, such ‘standardisation’ benefits must not be taken for granted, as different maintenance methods, other local differences and even accidental contamination from other colonies may lead to substantial differences between ‘identical’ laboratory stains [20^••^], and there are increasing calls for schemes to authenticate vector lines used in infection studies [21], similar to the authentications increasingly required for cell lines [22^•^].

As noted above, the increased potential for reproducibility afforded by the use of established insect colony lines may also come at the expense of reflecting the epidemiologically relevant diversity present in field populations. Natural ‘populations’ may be highly genetically heterogeneous, and even represent an assemblage of multiple cryptic species [23, 24, 25, 26, 27]. This has been a particular problem for understanding the epidemiology of *Culicoides*-borne viruses in Northern Europe, where the females of some vector species are morphologically cryptic [28] (e.g. *Culicoides obsoletus* (Meigen) and *Culicoides scoticus* Downes and Kettle [29]), confounding and limiting studies that do not use molecular identification techniques to resolve these issues [28,30]. The environments experienced by natural populations, including nutritional stresses, co-infection and thermal stresses, and which may change rapidly over short periods of time, will also not be represented without careful experimental design and pilot studies, for instance to establish a typical rate of natural co-infection [31, 32, 33]. Even when multiple vectors are known to be involved in transmission, difficulties in colonising some species may mean that subsets or proxies are used for the majority of studies. For example, within the *Culicoides* genus only two species are continuously maintained in colony globally: the North American BTV vector *Culicoides sonorensis* Wirth and Jones, and the European species *Culicoides nubeculosus* (Meigen) [34], which is typically refractory to infection. Attempts to develop colonies of other vector species of *Culicoides* have been unsuccessful to date [35]. As a consequence models of the transmission of BTV in Europe or Africa, where *Culicoides sonorensis* is absent, are based largely or solely on data obtained from infection studies conducted on a North American vector species. If obtaining isolates of relevant virus strains is problematic, alternative virus strains may also be used, potentially introducing similar issues.

## Maintenance of vectors

Studies can also be significantly affected by the methods used for insect maintenance, both before and after arbovirus exposure. The most important factors falling within this category include diet and temperature. The latter is the critical determinant of the duration of the extrinsic incubation period, and even small changes in temperature can have large consequences for *R*_0_ [36] but exposing larval vectors to higher temperatures can also affect competence [37,38]. The diets provided to *Aedes* and *Anopheles* mosquitoes as larvae have been shown to affect not only their rate of development but also their permissiveness to infection by arboviruses [38,39] and *Plasmodium* [40] respectively, and adult diet before pathogen exposure may also affect the microbiome [41], which could potentially alter the outcome of vector–pathogen interactions [42]. After exposure to a pathogen, the decision to maintain vectors on sugar solution or blood also significantly affects the outcome of the interaction [43^••^], a finding that has also been observed for *Leishmania* infection studies [44].

Other potential modulators of vector competence include the vector microbiome and virome, which may vary significantly between field and colony populations. However, co-infecting agents such as insect-specific viruses may persist cryptically within colonies for extended periods [45,46] leading to potentially confounding effects in comparative studies between laboratories and/or studies temporally separated but using the same colony line. Larval environment is a strong determinant of microbiome [47^•^] and differences in microbiome may significantly affect multiple aspects of development [48^•^]. Accordingly, researchers should consider microbiome and virome characterisation of insect colonies and field populations for co-infections that might affect the outcome of infection studies.

## Method of infection

The most epidemiologically relevant method of providing an infectious bloodmeal to potential vectors is to let them feed on a viraemic natural host [49]. However, there are obvious ethical reasons to conduct as few deliberate infections of human or animal hosts as possible. During such studies, infected vertebrates are normally maintained in containment both to prevent hazardous pathogens from escaping into the wider environment but also to prevent infections with pathogens that naturally circulate in the environment from affecting the outcome of the study. Such containment is often associated with extremely high operating costs. The short viraemia exhibited by some arboviruses may also make such experiments logistically difficult, and it is difficult to manipulate the dose to which a vector is exposed via natural feeding methods. More commonly, infectious bloodmeals are offered via artificial feeding systems, in which blood is typically provided via either membrane-covered reservoirs warmed to host body temperature (such as the now widely used Hemotek^®^ system (Hemotek Ltd, Blackburn, UK) (Figure 1)) or via pledgets of cotton wool or similar material soaked in blood-virus mixture. The choice of feeding system may affect the dose to which the vector is exposed and subsequent infection rates [50], this may be due to variations in the size of the bloodmeal taken or the destination of the bloodmeal within the vector — midgut or diverticulum [51]. The latter is a chitin-lined sac used for carbohydrate nutrient storage and typically refractory to infection [52]. Due to the small bloodmeal size taken by arbovirus vectors (typically ≤6 μl [53, 54, 55]), detecting subtle differences in bloodmeal sizes between feeding methods can be problematic.

To permit manipulation and prevent clotting, blood is normally defibrinated or mixed with an anti-coagulant such as heparin or EDTA. The influence of anti-coagulant on the outcome of vector–virus interactions is largely unknown, but some commonly used anticoagulants have been shown to significantly affect vector survival [56]. The dose of virus present in the bloodmeal will also affect the outcome of vector–virus interactions [57,58]. Logically, the most epidemiologically relevant studies would provide a bloodmeal at a host-equivalent titre. However, this may result in extremely low infection rates [59], and although these may be epidemiologically significant if balanced by high vector populations (as often seen for *Culicoides* [59]), they are of limited utility for the investigation of vector–virus interactions. In such situations unnaturally high bloodmeal titres may be required to yield useful infection rates. In addition, it should be remembered that the epidemiological relevance of a vector is not dictated solely by its competence but also factors such as host preference [60].

Other potential sources of variation include the origin of blood used in infection experiments. The species from which blood is sourced has been shown to strongly affect the outcome of vector–virus interactions, such as those between African horse sickness virus and *Culicoides sonorensis* [61] via the activity of host-species-specific serum proteases that cleave the outer capsid VP2 protein of the virus particle, which for the closely related *Orbivirus* BTV has been shown to increase the infectivity of virus particles for insect cells [58]. Other host blood-derived factors may also persist through digestion to affect vector lifespan, reproduction and immune response [62]. The presence of antibodies against relevant viral antigens in the bloodmeal may also affect the outcome of infection [63], however this potential effect has been characterised in only a limited number of systems. While sourcing ‘antibody-free’ blood should not be an issue in countries where transmission of the relevant virus(es) has not occurred, it may be problematic in countries where transmission is occurring and/or vaccination has been utilised.

Low feeding rates for some vectors, particularly field-caught vectors, coupled with the low dissemination rates described above, make the design of studies guaranteeing large numbers of insects developing transmissible infections (for example insect–host transmission studies requiring insects of known infection status, or studies investigating a small effect size) challenging. One solution often used is intrathoracic inoculation, in which virus is introduced directly into the thorax of the vector. The mortality associated with such a method is typically outweighed by the quantitative benefit of avoiding the problems of low feeding rates and highly effective midgut infection and escape barriers [64,65^•^], and may be useful for dissecting the relative roles of the dissemination barriers within the insect in determining vector competence [52] or as an initial screen of potential vectors [66], but given the route of infection and dose, the scope of research questions for which this approach can be considered useful is necessarily narrow.

The outcome of vector infection studies may be significantly affected by the virus source. When viral particles in the bloodmeal originate from cell culture, both the cell line used to propagate the virus and any secondary processing such as purification may impose selective pressures on the virus population, leading to significant effects on the genetic diversity of viral particles present in the bloodmeal. For example, Caporale *et al*. [67] found passage of BTV on mammalian cells (BHK-21, BSR, Vero and CPT-Tert) resulted in a significant reduction in genetic diversity compared to virus passaged on KC cells (a cell line derived from embryonic *C. sonorensis* [68]). A similar purifying selective effect during natural replication within the host compared to the vector has been identified for West Nile virus [69].

In addition to the vectors microbiome and virome, one last factor to consider within experimental design is the presence of co-infecting viruses, parasites and other agents within the infectious bloodmeal. Evidence to date indicates that the presence of parasites such as microfilarial nematodes is likely to be of greater importance than the presence of other arboviruses in a bloodmeal, with studies of simultaneous co-ingestion of multiple arboviruses resulting in similar levels of infection, dissemination and transmission to those seen during single-virus studies [70], suggesting that considering each virus in isolation for epidemiological purposes may be entirely valid. However, several studies have shown that simultaneous ingestion of an arbovirus and a nematode may result in elevated susceptibility to the arbovirus [71, 72, 73], initially assumed to be a result of the mechanical damage to the gut caused by the microfilariae allowing the virus to bypass the midgut infection and escape barriers in a similar way to intrathoracic inoculation, although more recent evidence has suggested that certain arboviruses may specifically adhere to the microfilariae and be actively transported within the insect [74]. Simulation studies suggest that this interaction could result in macro-scale epidemiological differences in outbreak frequency [75].

## Inference of transmissibility

Issues with inferring the infection status of vectors following exposure fall into two broad categories. The first is the decision to detect or quantify infectious virus or viral genome. The latter may detect not only infectious viral particles but also defective virus particles or RNA fragments, but the higher sensitivity of qPCR and related techniques may allow the detection of potentially transmissible infections which are below the detection threshold for cell culture based techniques [76]. In addition, different cell lines will have varying sensitivities to infection — often influenced by the passage history of the virus used [77]. Ultimately, the best method for a given study will depend on the research question, with limitations clearly identified within resulting publications.

The second is whether to collect saliva, which is time-consuming and problematic especially for small vector species, for example, *Culicoides* (body-size 1–3 mm), or to use a proxy such as the presence of virus in the salivary glands [78], in other parts of the body such as the head [14] or legs [79,80] assumed to indicate dissemination, or even to use whole-body thresholds [13] or pools of individuals [81].

## Conclusions and directions for future research

**Figure 2:**
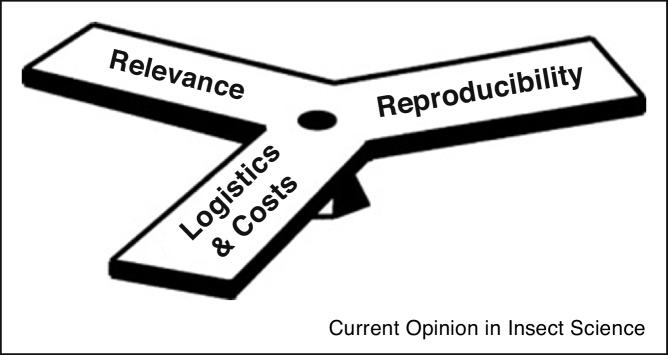
Illustration of the three considerations when choosing between alternative designs for insect-arbovirus infection studies.

The choice of techniques used in insect-infection studies is necessarily a balance between reproducibility, relevance and practicality (Figure 2), particularly for high containment studies. These studies are intrinsically difficult to reproduce due to the high cost and inflexibility of methods which are required to comply with health and safety legislation and hazard classification, which may vary between countries.

To increase confidence in the findings of such studies, we make several recommendations. Firstly, we recommend the publication of open-access protocols and raw data. This is increasingly required by publishers and facilitated by the availability of open access data repositories. Secondly, we recommend the routine screening, regular authentication and characterisation of cell lines, virus isolates and insect colonies used in insect infection studies, and reporting of these findings in relevant publications. Thirdly, we advocate more consistent measurement and reporting of factors which are known to strongly influence the outcome of insect exposure to virus. Given the volume of publications in the field, we suggest the most realistic way to ensure this last recommendation would be via the development, including regular review, by the vector community of reporting standards similar to those developed for quantitative PCR [82]. However, financial and technical constraints may limit the extent to which these recommendations can be followed. Ultimately, all methods have limitations, and the best design for a given study will depend on the research question, which must be clearly defined.

## Conflict of interest statement

Nothing declared.

## References and recommended reading

Papers of particular interest, published within the period of review, have been highlighted as

• of special interest
•• of outstanding interest
